# Function-Oriented Synthesis of Marine Phidianidine Derivatives as Potential PTP1B Inhibitors with Specific Selectivity

**DOI:** 10.3390/md16030097

**Published:** 2018-03-20

**Authors:** Jin Liu, Yu Chen, Jing-Ya Li, Cheng Luo, Jia Li, Kai-Xian Chen, Xu-Wen Li, Yue-Wei Guo

**Affiliations:** 1 State Key Laboratory of Drug Research, Shanghai Institute of Materia Medica, Chinese Academy of Sciences, 555 Zu Chong Zhi Road, Zhangjiang Hi-Tech Park, Shanghai 201203, China; ljin080@mail.ustc.edu.cn (J.L.); chenyu@simm.ac.cn (Y.C.); jyli@simm.ac.cn (J.-Y.L.); cluo@simm.ac.cn (C.L.); jli@simm.ac.cn (J.L.); kxchen@simm.ac.cn (K.-X.C.); 2 Nano Science and Technology Institute, University of Science and Technology of China, 166 Ren Ai Road, Suzhou 215123, China; 3 Open Studio for Druggability Research of Marine Natural Products, Qingdao National Laboratory for Marine Science and Technology, 1 Wenhai Road, Aoshanwei, Jimo, Qingdao 266237, China

**Keywords:** phidianidine, marine natural products, PTP1B inhibitor, specific selectivity, docking analysis, Function Oriented Synthesis, structure-activity relationship

## Abstract

Phidianidines A and B are two novel marine indole alkaloids bearing an uncommon 1,2,4-oxadiazole ring and exhibiting various biological activities. Our previous research showed that the synthesized phidianidine analogs had the potential to inhibit the activity of protein tyrosine phosphatase 1B (PTP1B), a validated target for Type II diabetes, which indicates that these analogs are worth further structural modification. Therefore, in this paper, a series of phidianidine derivatives were designed and rapidly synthesized with a function-oriented synthesis (FOS) strategy. Their inhibitory effects on PTP1B and T-cell protein tyrosine phosphatase (TCPTP) were evaluated, and several compounds displayed significant inhibitory potency and specific selectivity over PTP1B. The structure–activity relationship (SAR) and molecular docking analyses are also described.

## 1. Introduction

**Figure 1:**
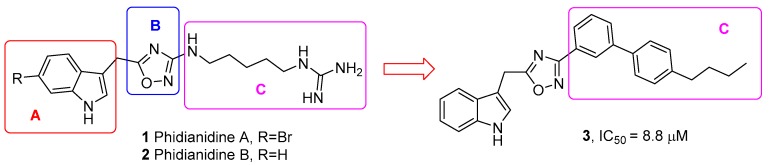
Structures of phidianidines (**1** and **2**) and their protein tyrosine phosphatase 1B (PTP1B) inhibitory analog **3**.

Protein tyrosine phosphatase 1B (PTP1B) is well-recognized as a potential target for the treatment of type II diabetes and obesity [1,2,3,4,5]. Many efforts have been made for the development of PTP1B inhibitors, while their low selectivity over the other protein tyrosine phosphatases (PTPs) and poor cell permeability are still two main issues, which prevent these compounds from being developed as marketed drugs [6,7]. Therefore, searching for new specifically selective PTP1B inhibitors is of high importance. In recent years, natural marine products have been regarded as new sources of potential PTP1B inhibitors, since various marine-derived PTP1B inhibitory phenols, terpenes, alkaloids, and terpene-alkaloid hybrids were isolated from algae, sponges, marine fungi, etc., with IC_50_ values ranging from 0.8 to 15 μM [8]. Our group has long been engaged in the discovery of bioactive natural marine products, with various novel molecules being isolated and structurally identified [9,10,11]. For example, phidianidines A and B (Figure 1) are two unprecedented indole alkaloids, bearing an uncommon 1,2,4-oxadiazole ring and a terminal guanidine group, isolated from the marine opisthobranch mollusk *Phidiana militaris* in 2011 [12]. These metabolites and their derivatives were found to exhibit significant cytotoxic, DAT inhibitory, or neuroprotective activities [13,14,15].

Based on previously mentioned bioactivities of phidianidines, in 2016 our group designed a series of new phidianidine analogs, and first reported their PTP1B inhibitory activities [16]. In this previous work, we assumed that the guanidine group was not the required function group, according to Lindersley’s research [14], and thus we did the preliminary function-oriented synthesis (FOS) of the phidianidine analogs towards PTP1B inhibitors. Several synthesized products (e.g., compound **3**, Figure 1) exhibited considerable inhibitory activities, with specific selectivity against other PTPs, such as T-cell protein tyrosine phosphatase (TCPTP); in addition, their synthesis is easy in comparison to the natural products with a guanidine group. However, there were still seven synthetic steps involved in the route, with transition metals needed in two of them, which are not economical and eco-friendly enough. Besides, whether the guanidine group is a required functionality or not for the PTP1B inhibitory effect is still not confirmed. Therefore, further FOS and structure–activity relationship (SAR) study are worthwhile, to be conducted towards more biologically-active yet affordable PTP1B inhibitors. It is worth mentioning that TCPTP, a phosphatase implicated in regulating T-cell activation, as a very important member of the PTP family, shows the highest homology to PTP1B. Therefore, selective inhibitory effect on PTP1B over TCPTP is essential for anti-diabetic drug discovery. In this paper, we prepared 40 phidianidine analogs (**9a**–**9e**, **10a**–**10e**, **13a**–**13i**, and **14a**–**14u**) with two different synthetic routes, by simplifying the **C** moiety on top of our previous result. The analogs’ PTP1B inhibitory activities were evaluated and the SAR was investigated. All the compounds were subjected to specific selectivity studies over TCPTP. A docking analysis of selected compounds **14c**, **14p** and **14l**−**14n** into the active site of PTP1B was also performed.

## 2. Results and Discussion

### 2.1. Initial Synthesis of Analogs and Biological Evaluation

**Scheme 1:**
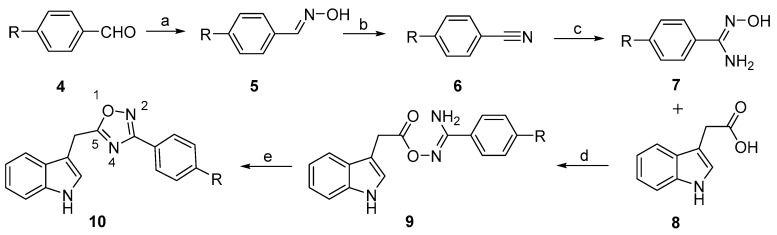
Reagent and conditions: (**a**) NH_2_OH·HCl, NaOH, 50% EtOH, r.t., overnight; (**b**) Dichloro(*p*-cymene)ruthenium(II) dimer, CH_3_CN, 80 °C, 4 h; (**c**) NH_2_OH·HCl, NaHCO_3_, EtOH, r.t., 4 h; (**d**) HATU, DIPEA, CH_2_Cl_2_, r.t., 2 h; (**e**) NaOAc, 30% EtOH, reflux, overnight.

The initial plan was to simplify the **C** moiety of compound **3** by removing one aryl ring, as shown in Scheme 1. The synthesis was similar as our previous reported route. The treatment of aryl aldehyde **4** (**4a**−**4d**) hydroxylamine hydrochloride (NH_2_OH·HCl), in the presence of sodium hydroxide (NaOH) in 50% EtOH (in water), yielded oxime **5**. Dehydration of **5** with a dichloro(*p*-cymene)ruthenium(II) dimer in acetonitrile (CH_3_CN) led to the nitrile **6** [17], which further reacted with NH_2_OH·HCl and sodium bicarbonate (NaHCO_3_) in EtOH, affording amidoxime **7**. Esterification of **7** with 3-indoleacetic acid in the presence of 2-(7-Azabenzotriazol-1-yl)-*N*,*N*,*N*′,*N*′-tetramethyluronium hexafluorophosphate (HATU) and *N*,*N*-diisopropylethylamine (DIPEA) in CH_2_Cl_2_ provided compound **9**. Intramolecular cyclization of **9** in the presence of sodium acetate (NaOAc) in 30% EtOH under reflux gave rise to the oxadiazole product **10** [18]. In order to make sure that the guanidine group was not correlated for the PTP1B inhibitory activity, the natural product phidianidine B (**2**) was also synthesized by following Chamberland’s route [19].

The inhibitory activities of the synthesized phidianidine B (**2**) and its analogs **9a**–**9e** and **10a**–**10e** against PTP1B were measured, using p-nitrophenyl phosphate (pNPP) as a substrate. Oleanolic acid, a known PTP1B inhibitor, was used as the positive control. The results are summarized in Table 1. The primary bioassay results indicated that the initial FOS is unsatisfied, since only one synthetic step was shortened, while just compound **10e** displayed moderate PTP1B inhibitory activity, with 50.5% inhibition (IC_50_ = 16.8 μM). Nevertheless, the preliminary SAR study suggested that the 1,2,4-oxadiazole was necessary for the activity, since the ring opening compounds **9a**−**9e** showed no effects. Moreover, the larger R group seemed to be helpful for the biological effect, as revealed by the inhibition percentage of **10c**−**10e**, while the guanidine chain was proved to be unwanted as showed by the inhibition rate of **2**.

### 2.2. Second Round Synthesis and Biological Evaluation

**Scheme 2:**
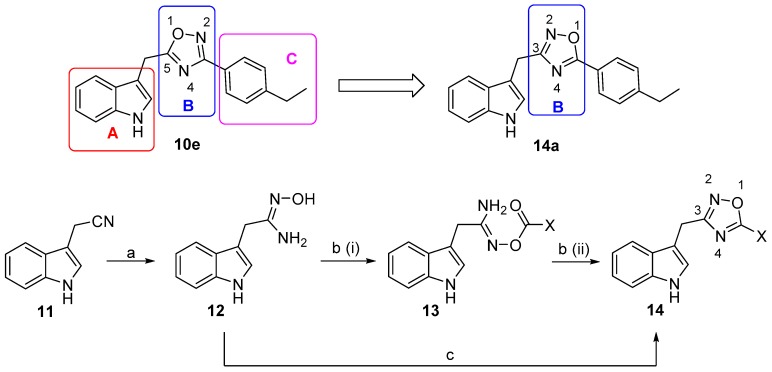
Reagent and conditions: (**a**) NH_2_OH·HCl, NaHCO_3_, EtOH, 65 °C, 4 h; (**b**) for **14a**–**14k**, and **14p**–**14u**: i, carboxylic acid, HATU, DIPEA, CH_2_Cl_2_, r.t., 2 h; ii, NaOAc, 30% EtOH, reflux, overnight. (**c**) For **14l**–**14o**, acyl chloride, K_2_CO_3_, toluene, reflux, 4 h.

In order to shorten the synthetic steps, and to efficiently obtain more analogs for further biological evaluation, we decided to change the linkage of the **A** and **B** moieties from the C-5 position of the 1,2,4-oxadiazole in compound **10** to its C-3 position in compound **14**, as displayed in Scheme 2, of which the synthetic steps could then be greatly reduced to three or two steps. With this strategy, we first synthesized compound **14a** as the aforementioned **B** moiety isomer of compound **10e**, and evaluated its PTP1B inhibitory activity. Fortunately, **14a** exhibited the similar effect as **10e**, with an IC_50_ value of 13.5 μM, which proved that the linkage position of the 1,2,4-oxadiazole has no obvious influence on the activity. Therefore, starting from 3-indoleacetonitrile (**11**), by using the synthetic routes shown in Scheme 2, a nucleophile was added, followed by esterification with either carboxylic acids or acyl chlorides and an immediate 1,2,4-oxadiazole ring closing under reflux, yielding a number of **14a** analogs **14b**–**14u**. It is worth mentioning that when treating compound **13i** in the presence of NaOAc under reflux in order to close the 1,2,4-oxadiazole ring, the bromine group has also been substituted by acetoxyl group towards compound **14i**.

In fact, compounds **14b**–**14u** were designed and synthesized in three directions. The first one was based on the previous results of **10e** and **14a**, of which different substitutes were introduced to the benzene ring of the **C** moiety, including nucleophilic and electrophilic groups. As indicated from the PTP1B inhibitory results of **14a**–**14h** in Table 2, the length of the alkyl side chain on the benzene ring played a highly important role on the activity. Compound **14c**, possessing the longest hexyl chain, exhibited the strongest PTP1B inhibitory activity, with an IC_50_ value of 4.9 μM. Other nucleophilic groups, such as methoxyl (**14f**), or an electrophilic group like trifluoromethyl (**14d**) showed no activity at 20 μM. The second direction was to replace the phenyl group with aliphatic and naphthenic substitutions towards **14h**–**14n**, of which the compounds comprising naphthenic groups such as cyclobutyl (**14l**) and cyclohexyl (**14n**) displayed significant PTP1B inhibitory activities, with IC_50_ values of 8.6 and 5.3 μM, respectively. The last route was to replace the phenyl group on other aromatic rings, such as pyridine and indole, towards **14o**–**14u**. As can be observed from the results in Table 2, the introduction of the pyridine ring (for **14o**) has no influence on improving inhibitory activity, while the indole ring greatly improved the effect, with IC_50_ values ranging from 5.8 to 9.7 μM for compounds **14p**–**14u**, among which **14p** (IC_50_ = 5.8 μM) is the strongest PTP1B inhibitor. The SAR analysis revealed that the presence of the halogen substitutions reduced inhibitory activity, with the F group shown to be the worst. In addition, the 1,2,4-oxadiazole group was further confirmed to be necessary for inhibitory activity, since the ring opening compounds **13a**−**13c** all showed no activity comparing to the corresponding **14a**−**14c**.

The selectivity of all the PTP1B inhibitory compounds was also evaluated against TCPTP. As shown in Table 3, none of the compounds showed significant inhibitory activities against TCPTP at the concentration of 20 μM, suggesting highly specific selectivity of these molecules towards PTP1B. Among them, compound **14n** showed the lowest inhibitory percentage at 20 μM against TCPTP, indicating the highest selectivity of **14n** on PTP1B.

### 2.3. Structure–Activity Relationship and Docking Analyses

**Figure 2:**
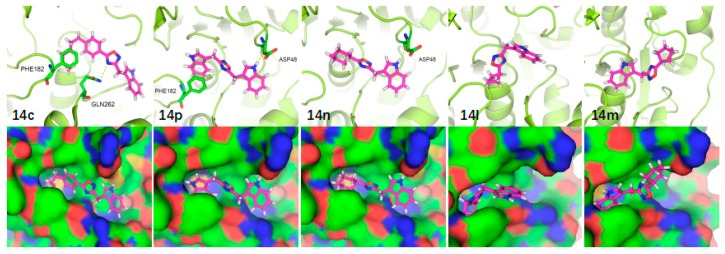
Docking results for compounds **14c**, **14p**, and **14l**–**14n** on PTP1B, respectively (upper row: without showing surface of PTP1B, lower row: showing surface of PTP1B).

In summary, the FOS of phidianidine analogs led to the discovery of three different PTP1B inhibitory compounds—**14c**, **14n**, and **14p**—with IC_50_ values of 4.9, 5.3, and 5.8 μM, respectively. From the structures of all the three compounds, and based on the preliminary SAR study, we speculate that the strong activity of compound **14c** might be due to the long chain well filling the ligand-binding pocket of the PTP1B protein. The improved activity of **14p** compared to the other aromatic analogs could possibly be attributed to the hydrogen bond interaction of its NH with the amino acid residue of PTP1B. However, the significant activity of the cyclohexyl-substituted compound **14n** was difficult to explain. On the basis of the above speculation, and in order to understand the inhibitory mechanism of the most active compounds against PTP1B, compounds **14c**, **14n**, and **14p** were initially selected to perform the molecular docking analysis [10]. The X-ray crystal structure of PTP1B, with a resolution of 2.50 Å (Protein Data Bank, 2QBP), was used for the docking studies. Figure 2 displays the binding mode of the selected compounds with PTP1B. For **14c**, we can easily observe the long chain filling the ligand-binding pocket of the PTP1B protein (Figure 2, docking figure of **14c** showing surface). Moreover, the alkyl chain on the benzene ring overlapped with the PHE182 amino acid residue, and the benzene ring helped to induce the alkyl chain into the right pocket; otherwise, the flexible long alkyl chain cannot fill the inner space, which reasonably explains the disappearance of the inhibitory activity for **14h** and **14i** (Figure 2). In addition, the N–H on the oxadiazole ring of **14c** formed a hydrogen bond interaction with the glutamate residues (GLN262), which enhanced its activity. For **14p** and **14n**, the N–H on the indole ring can form a hydrogen bond interaction with the aspartic acid residue (ASP48) of PTP1B. In addition, Figure 2 showed that the cyclohexyl of **14n** and the other indole ring of **14p** could fill the inner space of the pocket with the appropriate molecular size. However, it is still difficult to explain the lack of activity for compound **14m**, since its cyclopentyl moiety seems to have no big difference to the cyclohexyl on **14n**. Therefore, the **14n** analogs **14l** and **14m** were also applied for the docking analysis, and interestingly, **14m** showed totally different docking results from those of **14l** and **14n**, with the cyclopentane ring on the opposite site of the pocket (Figure 2). Besides, there were no bond interactions between **14m** and PTP1B, which further explained the disappearance of its inhibitory activity. For **14l**, although no bond interactions were found, its cyclobutane ring was small enough to go deeply into the pocket (Figure 2), which guaranteed the relatively strong activity. Finally, the molecular docking results rationally explained the PTP1B activity of the synthetic compounds and supported our SAR analysis. Compound **14n**, as the most selective candidate over PTP1B, should be further modified on its cyclohexane ring by adding a long alkyl chain and hydrogen bond donor to form a better interaction structure for binding to PTP1B with stronger activity and better selectivity. This research would thus give an insight on the discovery of novel specific PTP1B inhibitors from marine sources towards anti-diabetes drugs.

## 3. Experimental Section

### 3.1. Chemistry

All the chemicals were obtained from commercial sources. The NMR spectra were measured on Bruker Avance spectrometers (400 MHz for ^1^H; Avance III 400, and 125 MHz for ^13^C; Avance III 500, Bruker Biospin AG, Uster, Switzerland). Chemical shifts were expressed in δ (ppm) and coupling constants (*Ј*) in Hz. Commercial Silica gel (200–300 mesh, Sinopharm Chemical Reagent Co., Ltd., Shanghai, China) was used for column chromatography and pre-coated Silica gel plates (HSGF254, Sinopharm Chemical Reagent Co., Ltd., Shanghai, China) were used for analytical TLC. ESI-MS spectra were recorded on a Q-TOF Micromass spectrometer (1290-6545 UHPLC-QTOF, Micromass, Wythenshawe, UK).

#### 3.1.1. General Synthetic Procedure of Oxime ***5*** and Nitrile ***6***

To a solution of aldehyde **4** (9.4 mmol, 1.0 equiv) in EtOH (30 mL) was added hydroxylamine hydrochloride (11.3 mmol, 1.2 equiv) and sodium hydroxide (18.8 mmol, 2.0 equiv). The mixture was stirred at room temperature overnight. EtOH was removed in vacuo. The residue was added water and extracted with ethyl acetate (3 × 30 mL), washed with brine (3 × 30 mL), dried over anhydrous MgSO_4_, and concentrated. The residue was subjected to silica gel chromatography with petroleum ether/CH_2_Cl_2_ (3:2) to make oxime **5**. To a solution of oxime **5** (6.0 mmol, 1.0 equiv) in acetonitrile was added [Ru_2_(p-Pr^i^C_6_H_4_Me)_2_(μ-Cl)Cl]_2_ (0.3 mmol, 0.05 equiv) and refluxed for 4 h. The mixture was filtered, and the filtrate was concentrated in vacuo. The residue was subjected to silica gel chromatography with petroleum ether/CH_2_Cl_2_ (2:1) to make nitrile **6**.

#### 3.1.2. Synthesis of Carboxamidine **7**

To a solution of nitrile **6** (3.3 mmol, 1.0 equiv) in EtOH (15 mL) was added hydroxylamine hydrochloride (4.0 mmol, 1.2 equiv) and NaHCO_3_ (6.6 mmol, 2.0 equiv). The mixture was refluxed for 4 h. The reaction mixture was diluted with EtOAc, filtered, and concentrated in vacuo. Water was added to the residue and extracted with ethyl acetate (3 × 30 mL), washed with brine (1 × 30 mL), dried over anhydrous MgSO_4_, and concentrated. The residue was subjected to silica gel chromatography with EtOAc/MeOH (9:1) to make carboxamidine **7**.

#### 3.1.3. Synthesis of Carboxamidine **9**

To a solution of 3-indoleacetic acid (3.6 mmol, 1.0 equiv) in CH_2_Cl_2_ (20 mL), DIPEA (4.7 mmol, 1.3 equiv) and HATU (3.6 mmol, 1.0 equiv) were added, and the reaction mixture was stirred for 30 min, then carboxamidine **7** (3.6 mmol, 1.0 equiv) dissolved in CH_2_Cl_2_ (10 mL) was added and stirred for 2 h. The mixture was filtered and the residue was washed with CH_2_Cl_2_, after which the solution was combined and concentrated. The residue was subjected to silica gel chromatography with petroleum ether/EtOAc (2:1) to give carboxamidine **9**.

**9a**: White solid, Yield 85%; ^1^H NMR (400 MHz, CD_3_OD): δ 8.13 (d, *J* = 7.15 Hz, 1H),7.70 (d, *J* = 7.78 Hz, 1H), 7.61 (d, *J* = 7.45 Hz, 1H), 7.50 (t, *J* = 7.66 Hz, 2H), 7.35 (d, *J* = 8.12 Hz, 1H), 7.27 (s, 1H), 7.11 (t, 1H), 7.03 (t, 1H), 3.73(s, 2H); ^13^C NMR (125 MHz, CD_3_OD): δ 166.42, 161.34, 138.25, 134.25, 130.70, 130.55, 129.64, 128.49, 125.03, 122.67, 120.01, 119.63, 112.29, 109.59, 28.25; HR-ESIMS: [M + H]^+^ calcd. for C_17_H_16_N_3_O_2_ 294.1237, found: 294.1231.

**9b**: White solid, Yield 80%; ^1^H NMR (400 MHz, CD_3_OD): δ 7.75 (m, 2H), 7.64 (d, 1H), 7.37 (d, 1H), 7.25 (s, 1H), 7.15 (m, 3H), 7.05 (t, 1H), 3.96(s, 2H); ^13^C NMR (125 MHz, CD_3_OD): δ 172.08, 164.41, 158.89, 137.69, 130.55, 130.46, 128.58, 127.79, 124.95, 122.60, 120.03, 119.51, 116.54, 116.32, 112.41, 108.40, 30.80; HR-ESIMS: [M + H]^+^ calcd. for C_17_H_15_FN_3_O_2_ 312.1143, found: 312.1144.

**9c**: White solid, Yield 85%; ^1^H NMR (400 MHz, CD_3_OD): δ 7.70 (d, *J* = 8.79 Hz, 2H), 7.62 (d, *J* = 6.72 Hz, 1H), 7.43 (d, *J* = 8.79 Hz, 2H), 7.36 (d, *J* = 8.10 Hz, 1H), 7.25 (s, 1H), 7.11 (t, 1H), 7.06 (t, 1H), 3.96 (s, 2H); ^13^C NMR (125 MHz, CD_3_OD): δ 172.08, 158.85, 138.08, 137.85, 131.44, 129.81, 129.74, 129.57, 128.81, 128.61, 124.84, 122.58, 120.02, 119.42, 112.38, 108.37, 30.91; HR-ESIMS: [M − H]^−^ calcd. for C_17_H_13_ClN_3_O_2_ 326.0702, found: 326.0705.

**9d**: White solid, Yield 90%; ^1^H NMR (400 MHz, CD_3_OD): δ 8.28 (d, *J* = 9.07 Hz, 2H), 7.96 (d, *J* = 9.07 Hz, 2H), 7.64 (d, *J* = 7.92 Hz, 1H), 7.37 (d, *J* = 8.06 Hz, 1H), 7.26 (s, 1H), 7.12 (t, 1H), 7.06 (t, 1H), 3.98 (s, 2H); ^13^C NMR (125 MHz, CD_3_OD): δ 172.00, 157.92, 150.69, 138.90, 138.09, 129.53, 124.87, 124.56, 122.60, 120.03, 119.41, 112.40, 108.29, 30.85; HR-ESIMS: [M − H]^−^ calcd. for C_17_H_13_N_4_O_4_ 337.0942, found: 337.0952.

**9e**: White solid, Yield 85%; ^1^H NMR (400 MHz, CD_3_OD): δ 7.63 (m, 3H), 7.36 (d, 1H), 7.26 (m, 3H), 7.12 (t, 1H), 7.05 (t, 1H), 3.96 (s, 2H), 2.67 (m, 2H), 1.23 (t, 3H); ^13^C NMR (125 MHz, CD_3_OD): δ 172.15, 159.95, 148.71, 138.09, 130.05, 129.03, 128.63, 128.21, 124.82, 122.58, 120.02, 119.43, 112.37, 108.46, 30.97, 29.68, 15.89; HR-ESIMS: [M + H]^+^ calcd. for C_19_H_20_N_3_O_2_ 322.1550, found: 322.1543.

#### 3.1.4. Synthesis of Compound **10**

A solution of carboxamidine **9** (3.2 mmol, 1.0 equiv) and sodium acetate (6.4 mmol, 2.0 equiv) in 30% EtOH/H_2_O (10 mL) was refluxed overnight. The EtOH was removed in vacuo, and the residue was added to water and extracted with ethyl acetate (3 × 30 mL), then washed with brine (1 × 30 mL), dried over anhydrous MgSO_4_, and concentrated. The residue was subjected to silica gel chromatographic with petroleum ether/EtOAc (5:1) to make compound **10**.

**10a**: White solid, Yield 94%; ^1^H NMR (400 MHz, CD_3_OD): δ 8.10 (d, *J* = 7.05 Hz, 1H), 7.63 (d, 2H), 7.56 (d, 2H), 7.35(d, *J* = 8.13 Hz, 1H), 7.23 (s, 1H), 7.10 (t, 1H), 7.01 (t, 1H), 4.27(s, 2H); ^13^C NMR (125 MHz, CD_3_OD): δ 177.04, 172.0, 138.17, 134.06, 130.36, 129.02, 128.41, 125.39, 124.55, 122.59, 119.91, 119.37, 112.33, 109.82, 23.39; HR-ESIMS: [M + H]^+^ calcd. for C_17_H_14_N_3_O 276.1131, found: 276.1125.

**10b**: White solid, Yield 92%; ^1^H NMR (400 MHz, CD_3_OD): δ 8.08 (d, 1H), 8.06 (d, 1H), 7. 58 (d, *J* = 7.92 Hz, 1H), 7.37 (d, *J* = 8.14 Hz, 1H), 7.28 (s, 1H), 7.23 (t, 2H), 7.12 (t, 1H), 7.03 (t, 1H),4.46(s, 2H); ^13^C NMR (125 MHz, CD_3_OD): δ 181.15, 168.75, 164.99, 138.12, 130.73, 130.66, 128.16, 124.80, 124.60, 122.79, 120.19, 119.16, 117.09, 116.91, 112.46, 108.06, 24.01; HR-ESIMS: [M − H]^−^ calcd. for C_17_H_11_FN_3_O 292.0892, found: 292.0886.

**10c**: White solid, Yield 92%; ^1^H NMR (400 MHz, CD_3_OD): δ 8.01 (d, *J* = 8.75 Hz, 2H), 7.92 (d, *J* = 7.92 Hz, 1H), 7. 50 (d, *J* = 8.74 Hz, 2H), 7.37 (d, *J* = 8.14 Hz, 1H), 7.28 (s, 1H), 7.12 (t, 1H), 7.04 (t, 1H), 4.47 (s, 2H); ^13^C NMR (125 MHz, CD_3_OD): δ 181.27, 168.77, 164.99, 138.34, 138.12, 130.27, 129.86, 128.16, 126.92, 124.79, 122.79, 120.19, 119.15, 112.46, 108.03, 24.02; HR-ESIMS: [M − H]^−^ calcd. for C_17_H_11_ClN_3_O 308.0596, found: 308.0596.

**10d**: White solid, Yield 90%; ^1^H NMR (400 MHz, dimethyl sulfoxide (DMSO)-*d*_6_, not soluble in MeOH or CHCl_3_): δ 8.38 (d, *J* = 8.98 Hz, 2H), 8.24 (d, *J* = 8.98 Hz, 2H), 7.57 (d, *J* = 7.80 Hz, 1H), 7.43 (s, 1H), 7.39 (d, *J* = 8.11 Hz, 1H), 7.11 (t, 1H), 7.02 (t, 1H), 4.56 (s, 2H); ^13^C NMR (125 MHz, DMSO-*d*_6_): δ 180.31, 166.43, 149.13, 136.17, 132.05, 128.39, 126.61, 124.49, 124.44, 121.37, 118.86, 118.19, 111.63, 106.21, 22.76; HR-ESIMS: [M − H]^−^ calcd. for C_17_H_11_N_4_O_3_ 319.0837, found: 319.0835.

**10e**: White solid, Yield 85%; ^1^H NMR (400 MHz, CD_3_OD): δ 8.55 (t, 2H), 8.20 (t, 1H), 7.94 (m, 4H), 7.73 (t, 1H), 7.66 (t, 1H), 5.07 (m, 2H), 3.93 (s, 2H), 1.87 (m, 3H); ^13^C NMR (125 MHz, CD_3_OD): δ 180.87, 169.54, 149.26, 138.12, 129.47, 128.40, 128.17, 125.52, 124.78, 122.78, 120.18, 118.17, 112.45, 108.11, 29.79, 24.03, 15.82; HR-ESIMS: [M + H]^+^ calcd. for C_19_H_18_N_3_O 304.1444, found: 304.1450.

#### 3.1.5. Synthesis of Carboxamidine **12**

To a solution of compound **11** (64.1 mmol, 1.0 equiv) in EtOH (60 mL) was added hydroxylamine hydrochloride (96.2 mmol, 1.5 equiv) and NaHCO_3_ (192.3 mmol, 3.0 equiv). The mixture was stirred for 4 h at 65 °C. The reaction mixture was diluted with EtOAc, filtered and concentrated in vacuo. Water was added to the residue and extracted with ethyl acetate (3 × 100 mL), washed with brine (1 × 100 mL), dried over anhydrous MgSO_4_, and concentrated. The residue was subjected to silica gel chromatography with EtOAc /MeOH (9:1) to make carboxamidine **12**.

#### 3.1.6. Synthesis of Carboxamidine **13**

To a solution of carboxylic acid (0.8 mmol, 1.0 equiv) in CH_2_Cl_2_ (5 mL), DIPEA (1.0 mmol, 1.3 equiv) and HATU (0.8 mmol, 1.0 equiv) were added, and the reaction mixture was stirred for 30 min, after which compound **7** (0.8 mmol, 1.0 equiv) in CH_2_Cl_2_ (2 mL) was added and stirred for 2 h. The mixture was filtered, the residue was washed with CH_2_Cl_2_, and then the solution was combined and concentrated. The residue was subjected to silica gel chromatography with petroleum ether/EtOAc (2:1) to give **13**.

**13a**: White solid, Yield 88%; ^1^H NMR (400 MHz, CD_3_OD): δ 8.04 (d, 2H), 7.70 (d, 1H), 7.34 (m, 3H), 7.27 (s, 1H), 7.11 (t, 1H), 7.03 (t, 1H), 3.72 (s, 2H), 2.72 (q, 2H), 1.25 (t, 3H); ^13^C NMR (125 MHz, CD_3_OD): δ 166.44, 161.22, 151.46, 138.26, 130.74, 129.13, 125.01, 122.66, 120.01, 119.66, 112.28, 109.65, 29.89, 28.26, 15.73; HR-ESIMS: [M + H]^+^ calcd. for C_19_H_20_N_3_O_2_ 322.1550, found: 322.1555.

**13b**: White solid, Yield 82%; ^1^H NMR (400 MHz, CD_3_OD): δ 8.03 (d, 2H), 7.70 (d, 1H), 7.35 (d, 1H), 7.31 (d, 2H), 7.26 (s, 1H), 7.11 (t, 1H), 7.02 (t, 1H), 3.72 (s, 2H), 2.69 (t, 2H), 1.61 (m, 2H), 1.37 (m, 2H), 0.94 (t, 3H); ^13^C NMR (125 MHz, CD_3_OD): δ 166.45, 161.22, 150.08, 138.25, 130.64, 129.69, 125.01, 122.66, 120.01, 119.65, 112.28, 109.64, 36.62, 34.54, 28.26, 23.32, 14.20; HR-ESIMS: [M + H]^+^ calcd. for C_2__1_H_2__4_N_3_O_2_ 350.1863, found: 350.1870.

**13c**: White solid, Yield 80%; ^1^H NMR (400 MHz, CD_3_OD): δ 8.03 (d, 2H), 7.70 (d, 1H), 7.35 (d, 1H), 7.30 (d, 2H), 7.26 (s, 1H), 7.11 (t, 1H), 7.02 (t, 1H), 3.72 (s, 2H), 2.68 (t, 2H), 1.64 (m, 2H), 1.32 (m, 6H), 0.89 (t, 3H); ^13^C NMR (125 MHz, CD_3_OD): δ 166.46, 161.22, 150.10, 138.25, 130.64, 129.69, 125.01, 122.66, 120.01, 119.65, 112.28, 109.64, 36.92, 32.81, 32.31, 29.99, 28.26, 23.63, 14.37; HR-ESIMS: [M + H]^+^ calcd. for C_23_H_28_N_3_O_2_ 378.2176, found: 378.2182.

**13d**: White solid, Yield 85%; ^1^H NMR (400 MHz, CD_3_OD): δ 8.01 (t, 1H), 7.69 (d, 1H), 7.61 (m, 1H), 7.35 (d, 1H), 7.29 (t, 2H), 7.26 (s, 1H), 7.22 (t, 1H), 7.11 (t, 1H), 7.04 (t, 1H), 3.72 (s, 2H); ^13^C NMR (125 MHz, CD_3_OD): δ 164.04, 164.01, 161.96, 161.55, 138.23, 136.05, 135.98, 133.15, 128.46, 125.57, 125.54, 125.03, 122.67, 120.02, 119.60, 119.11, 119.03, 118.03, 117.85, 112.30, 109.45, 28.02; HR-ESIMS: [M + H]^+^ calcd. for C_17_H_15_FN_3_O_2_ 312.1143, found: 312.1144.

**13e**: White solid, Yield 85%; ^1^H NMR (400 MHz, CD_3_OD): δ 8.32 (d, *J* = 8.14 Hz, 2H), 7.81 (d, *J* = 8.28 Hz, 2H), 7.70 (d, *J* = 7.91 Hz,1H), 7.35 (d, *J* = 8.14 Hz, 1H), 7.27 (s, 1H), 7.11 (t, 1H), 7.02 (t, 1H), 3.73 (s, 2H); ^13^C NMR (125 MHz, CD_3_OD): δ 165.01, 161.67, 138.26, 135.23, 134.42, 131.27, 131.17, 128.48, 126.60, 126.57, 125.03, 122.67, 120.01, 119.65, 112.29, 109.59, 28.25; HR-ESIMS: [M + H]^+^ calcd. for C_18_H_15_F_3_N_3_O_2_ 362.1111, found: 362.1112.

**13f**: White solid, Yield 85%; ^1^H NMR (400 MHz, CD_3_OD): δ 8.08 (d, *J* = 8.97 Hz, 2H), 7.70 (d, *J* = 7.92 Hz, 1H), 7.35 (d, *J* = 8.12 Hz,1H), 7.26 (s, 1H), 7.11 (t, *J* = 7.56 Hz, 1H), 7.04 (s, 1H), 6.99 (t, *J* = 9.00 Hz, 1H), 3.85 (s, 2H), 3.71 (s, 3H); ^13^C NMR (125 MHz, CD_3_OD): δ 166.23, 165.19, 161.09, 138.25, 132.63, 128.50, 125.00, 122.78, 122.66, 120.00, 119.66, 114.88, 112.28, 109.66, 55.99, 28.27; HR-ESIMS: [M + H]^+^ calcd. for C_18_H_18_N_3_O_3_ 324.1343, found: 324.1347.

**13g**: White solid, Yield 80%; ^1^H NMR (400 MHz, CD_3_OD): δ 7.69 (d, 1H), 7.56 (d, 1H), 7.35 (d, 1H), 7.33 (d, 1H), 7.26 (s, 1H), 7.11 (t, 1H), 7.01 (m, 2H), 3.81 (s, 3H), 3.71 (s, 2H); ^13^C NMR (125 MHz, CD_3_OD): δ 166.11, 161.55, 160.37, 138.24, 135.87, 134.49, 128.47, 125.04, 122.67, 120.03, 119.80, 119.63, 117.40, 112.29, 111.77 109.46, 56.24, 28.05; HR-ESIMS: [M + H]^+^ calcd. for C_18_H_17_BrN_3_O_3_ 402.0448, found: 402.0445.

**13h**: White solid, Yield 85%; ^1^H NMR (400 MHz, CD_3_OD): δ 7.64 (d, 1H), 7.34 (d, 1H), 7.21 (s, 1H), 7.10 (t, 1H), 7.01 (t, 1H), 3.63 (s, 2H), 2.43 (t, 2H), 1.65 (m, 2H), 1.40 (m, 2H), 0.94 (t, 3H); ^13^C NMR (125 MHz, CD_3_OD): δ 173.57, 160.85, 138.21, 128.45, 124.93, 122.62, 119.93, 119.63, 112.25, 109.63, 33.38, 28.23, 28.12, 23.30, 14.07; HR-ESIMS: [M + H]^+^ calcd. for C_15_H_20_N_3_O_2_ 274.1550, found: 274.1553.

**13i**: White solid, Yield 80%; ^1^H NMR (400 MHz, CD_3_OD): δ 7.64 (d, 1H), 7.34 (d, 1H), 7.22 (s, 1H), 7.10 (t, 1H), 7.00 (t, 1H), 3.63 (s, 2H), 3.47 (t, 2H), 2.47 (t, 2H), 1.91 (m, 2H), 1.82 (m, 2H); ^13^C NMR (125 MHz, CD_3_OD): δ 173.07, 160.95, 138.21, 128.44, 124.93, 122.62, 119.94, 119.63, 112.25, 109.63, 33.72, 33.23, 32.63, 28.12, 24.65; HR-ESIMS: [M + H]^+^ calcd. for C_15_H_19_BrN_3_O_2_ 352.0655, found: 352.0663.

#### 3.1.7. Synthesis of Compound **14**

A solution of compound **9** (0.4 mmol, 1.0 equiv) and sodium acetate (0.8 mmol, 2.0 equiv) in 30% EtOH/H_2_O (5 mL), was refluxed overnight. The EtOH was removed in vacuo, the residue was added with water and extracted with ethyl acetate (3 × 5 mL), washed with brine (1 × 5 mL), dried over anhydrous MgSO_4_, and concentrated. The residue was subjected to silica gel chromatography with petroleum ether/EtOAc (5:1) to give compound **14**.

**14a**: White solid, Yield 84%; ^1^H NMR (400 MHz, CD_3_OD): δ 7.99 (d, 2H), 7.59 (d, 1H), 7.37 (d, 2H), 7.34 (d, 1H), 7.22 (s, 1H), 7.09 (t, 1H), 7.01 (t, 1H), 4.24 (s, 2H), 2.71 (q, 2H), 1.25 (t, 3H); ^13^C NMR (125 MHz, CD_3_OD): δ 177.14, 171.85, 151.36, 138.15, 129.83, 129.13, 124.53, 122.57, 119.90, 119.37, 112.32, 109.84, 29.89, 23.38, 16.63; HR-ESI: [M + H]^+^ calcd. for C_19_H_18_N_3_O 304.1444, found: 304.1447.

**14b**: White solid, Yield 80%; ^1^H NMR (400 MHz, CD_3_OD): δ 7.98 (d, 2H), 7.59 (d, 1H), 7.34 (m, 3H), 7.22 (s, 1H), 7.09 (t, 1H), 7.02 (t, 1H), 4.25 (s, 2H), 2.68 (t, 2H), 1.61 (m, 2H), 1.36 (m, 2H), 0.94 (t, 3H); ^13^C NMR (125 MHz, CD_3_OD): δ 177.15, 171.85, 150.01, 138.15, 130.39, 129.05, 124.53, 122.58, 119.90, 119.37, 112.32, 109.84, 36.62, 34.47, 23.39, 23.32, 14.19; HR-ESI: [M + H]^+^ calcd. for C_21_H_22_N_3_O 332.1757, found: 332.1754.

**14c**: White solid, Yield 86%; ^1^H NMR (400 MHz, CD_3_OD): δ 7.97 (d, 2H), 7.59 (d, 1H), 7.34 (m, 3H), 7.21 (s, 1H), 7.09 (t, 1H), 7.00 (t, 1H), 4.24 (s, 2H), 2.65 (t, 2H), 1.61 (m, 2H), 1.31 (m, 6H), 0.88 (t, 3H); ^13^C NMR (125 MHz, CD_3_OD): δ 177.13, 171.83, 150.00, 138.14, 130.36, 129.03, 124.53, 122.57, 119.90, 119.37, 112.32, 109.84, 36.91, 32.79, 32.23, 29.98, 23.61, 23.29, 14.37; HR-ESI: [M + H]^+^ calcd. for C_23_H_2__6_N_3_O 360.2070, found: 360.2073.

**14d**: White solid, Yield 82%; ^1^H NMR (400 MHz, CD_3_OD): δ 8.09 (t, 1H), 7.63 (m, 2H), 7.35 (m, 3H), 7.23 (s, 1H), 7.09 (t, 1H), 7.01 (t, 1H), 4.29 (s, 2H); ^13^C NMR (125 MHz, CD_3_OD): δ 174.00, 171.73, 163.05, 160.99, 138.14, 136.20, 136.13, 131.93, 128.39, 126.10, 126.08, 124.57, 122.58, 119.91, 119.39, 118.19, 118.02, 113.76, 112.32, 109.79, 23.32; HR-ESI: [M + H]^+^ calcd. for C_17_H_13_FN_3_O 294.1037, found: 294.1035.

**14e**: White solid, Yield 91%; ^1^H NMR (400 MHz, CD_3_OD): δ 8.27 (d, *J* = 8.16 Hz, 2H), 7.86 (d, *J* = 8.27 Hz, 2H), 7.60 (s, 2H), 7.34 (d, *J* = 8.13 Hz, 1H), 7.23 (s, 1H), 7.09 (t, *J* = 7.58 Hz, 1H), 7.01 (t, *J* = 7.50 Hz, 1H), 4.28 (s, 2H); ^13^C NMR (125 MHz, CD_3_OD): δ 175.67, 172.34, 138.15, 135.23, 134.97, 129.72, 128.88, 128.38, 127.32, 127.29, 126.17, 124.59, 124.00, 122.60, 119.93, 119.36, 112.34, 109.72, 23.36; HR-ESIMS: [M + H]^+^ calcd. for C_18_H_13_F_3_N_3_O 344.1005, found: 344.1010.

**14f**: White solid, Yield 90%; ^1^H NMR (400 MHz, CD_3_OD): δ 8.04 (d, 2H), 7.59 (d, 1H), 7.34 (d, 1H), 7.22 (s, 1H), 7.09 (m, 2H), 7.01 (t, 2H), 4.23 (s, 2H), 3.88 (s, 3H); ^13^C NMR (125 MHz, CD_3_OD): δ 176.99, 171.76, 165.01, 138.17, 131.00, 128.42, 124.53, 122.57, 119.90, 119.37, 117.66, 115.75, 112.32, 109.88, 56.10, 23.38; HR-ESI: [M + H]^+^ calcd. for C_18_H_16_N_3_O_2_ 306.1237, found: 306.1234.

**14g**: White solid, Yield 90%; ^1^H NMR (400 MHz, CD_3_Cl, in methanol dissolved is not good): δ 7.76 (d, 1H), 7.60 (d, 1H), 7.46 (d, 1H), 7.36 (m, 1H), 7.21 (m, 2H), 7.14 (t, 1H), 6.94 (dd, 1H), 4.34 (s, 2H), 3.82 (s, 3H); ^13^C NMR (125 MHz, CD_3_Cl): δ 174.77, 170.16, 158.86, 136.37, 135.62, 127.25, 126.40, 123.14, 122.46, 120.00, 119.84, 119.23, 116.71, 112.51, 111.31, 110.04, 55.89, 22.91; HR-ESI: [M + H]^+^ calcd. for C_18_H_15_BrN_3_O_2_ 384.0342, found: 384.0338.

**14h**: White solid, Yield 80%; ^1^H NMR (400 MHz, CD_3_OD): δ 7.51 (d, 1H), 7.33 (d, 1H), 7.16 (s, 1H), 7.08 (t, 1H), 6.98 (t, 1H), 4.15 (s, 2H), 2.84 (t, 2H), 1.72 (m, 2H), 1.35 (m, 2H), 0.91 (t, 3H); ^13^C NMR (125 MHz, CD_3_OD): δ 181.73, 171.02, 138.12, 128.32, 124.46, 122.54, 119.85, 119.30, 112.29, 109.79, 29.59, 26.83, 23.18, 23.06, 13.80; HR-ESI: [M + H]^+^ calcd. for C_15_H_18_N_3_O 256.1372, found: 256.1377.

**14i**: White solid, Yield 65%; ^1^H NMR (400 MHz, CD_3_OD): δ 7.51 (d, 1H), 7.33 (d, 1H), 7.17 (s, 1H), 7.09 (t, 1H), 6.98 (t, 1H), 4.17 (s, 2H), 4.06 (t, 2H), 2.91 (t, 2H), 2.00 (s, 3H), 1.85 (m, 2H), 1.69 (m, 2H); ^13^C NMR (125 MHz, CD_3_OD): δ 181.33, 172.92, 171.12, 138.14, 128.34, 124.48, 122.55, 119.86, 119.32, 112.30, 109.79, 64.90, 28.92, 26.70, 24.09, 23.20, 20.74; HR-ESI: [M + H]^+^ calcd. for C_17_H_20_N_3_O_3_ 314.1499, found: 314.1500.

**14j**: White solid, Yield 70%; ^1^H NMR (400 MHz, CD_3_OD): δ 7.53 (d, 1H), 7.33 (d, 1H), 7.16 (s, 1H), 7.08 (t, 1H), 6.99 (t, 1H), 4.15 (s, 2H), 3.17 (m, 1H), 1.32 (d, 6H), 1.35 (m, 2H), 0.91 (t, 3H); ^13^C NMR (125 MHz, CD_3_OD): δ 185.49, 170.96, 138.10, 128.33, 124.47, 122.54, 119.86, 119.31, 112.29, 109.77, 28.54, 23.22, 20.32; HR-ESI: [M + H]^+^ calcd. for C_14_H_16_N_3_O 242.1215, found: 242.1212.

**14k**: White solid, Yield 65%; ^1^H NMR (400 MHz, CD_3_OD): δ 7.55 (d, 1H), 7.35 (m, 2H), 7.19 (s, 1H), 7.10 (t, 1H), 7.00 (t, 1H), 4.24 (s, 2H); ^13^C NMR (125 MHz, CD_3_OD): δ 175.37, 172.18, 138.08, 128.24, 124.62, 122.62, 119.96, 119.26, 112.34, 109.19, 59.86, 23.21; HR-ESI: [M + H]^+^ calcd. for C_12_H_10_Cl_2_N_3_O 282.0123, found: 282.0127.

**14p**: White solid, Yield 65%; ^1^H NMR (400 MHz, CD_3_OD): δ 7.50 (d, 1H), 7.45 (d, 1H), 7.33 (dd, 2H), 7.17 (s, 1H), 7.13 (s, 1H), 7.09 (m, 2H), 6.97 (t, tH), 4.32 (s, 2H), 4.14 (s, 2H); ^13^C NMR (125 MHz, CD_3_OD): δ 178.67, 169.88, 136.35, 136.24, 123.12, 122.56, 122.35, 120.02, 119.76, 119.76, 119.06, 118.76, 111.42, 111.32, 109.82, 107.95, 23.47, 22.78; HR-ESI: [M + H]^+^ calcd. for C_20_H_17_N_4_O 329.1397, found: 329.1388.

**14q**: White solid, Yield 74%; ^1^H NMR (400 MHz, CD_3_OD): δ 7.50 (d, 1H), 7.29 (m, 2H), 7.20 (s, 1H), 7.16 (d, 1H), 7.12 (s, 1H), 7.07 (t, 1H), 6.97 (t, 1H), 6.87 (t, 1H) 4.25 (s, 2H), 4.13 (s, 2H); ^13^C NMR (125 MHz, CD_3_OD): δ 180.53, 171.21, 159.80, 158.26, 138.07, 134.57, 128.43, 128.36, 128.29, 126.73, 124.46, 122.54, 119.89, 119.31, 113.29, 113.22, 112.27, 110.98, 110.80, 109.74, 108.29, 108.25, 103.96, 103.80, 23.74, 23.17; HR-ESIMS: [M − H]^−^ calcd. for C_20_H_1__4_FN_4_O 345.1157, found: 345.1161.

**14r**: White solid, Yield 78%; ^1^H NMR (400 MHz, CD_3_OD): δ 7.51 (m, 2H), 7.31 (m, 2H), 7.24 (s, 1H), 7.14 (s, 1H), 7.08 (m, 2H), 6.97 (t, 1H), 4.31 (s, 2H), 4.16 (s, 2H); ^13^C NMR (125 MHz, CD_3_OD): δ 180.47, 171.27, 138.11, 136.45, 129.22, 128.33, 126.51, 125.99, 124.46, 122.94, 122.54, 119.90, 119.31, 118.69, 113.67, 112.28, 109.77, 108.00, 23.69, 23.19; HR-ESIMS: [M − H]^−^ calcd. for C_20_H_1__4_ClN_4_O 361.0862, found: 361.0858.

**14s**: White solid, Yield 82%; ^1^H NMR (400 MHz, CD_3_OD): δ 7.66 (d, 1H), 7.51 (d, 1H), 7.32 (d, 1H), 7.20 (m, 2H), 7.14 (s, 1H), 7.08 (t, 1H), 6.98 (t, 1H), 4.30 (s, 2H), 4.15 (s, 2H); ^13^C NMR (125 MHz, CD_3_OD): δ 180.44, 171.26, 138.11, 136.69, 129.88, 128.33, 126.35, 125.54, 124.46, 122.54, 121.84, 119.91, 119.31, 114.10, 113.38, 112.28, 109.76, 107.90, 23.67, 23.18; HR-ESIMS: [M − H]^−^ calcd. for C_20_H_1__4_BrN_4_O 405.0356, found: 405.0356.

**14t**: White solid, Yield 88%; ^1^H NMR (400 MHz, CD_3_OD): δ 7.51 (m, 2H), 7.31 (m, 2H), 7.24 (s, 1H), 7.14 (s, 1H), 7.08 (m, 2H), 6.97 (t, 1H), 4.31 (s, 2H), 4.16 (s, 2H); ^13^C NMR (125 MHz, CD_3_OD): δ 180.47, 171.27, 138.11, 136.45, 129.22, 128.33, 126.51, 125.99, 124.46, 122.94, 122.54, 119.90, 119.31, 118.69, 113.67, 112.28, 109.77, 108.00, 23.69, 23.19; HR-ESIMS: [M − H]^−^ calcd. for C_20_H_1__4_ClN_4_O 361.0862, found: 361.0870.

**14u**: White solid, Yield 82%; ^1^H NMR (400 MHz, CD_3_OD): δ 7.49 (d, 1H), 7.29 (t, 2H), 7.21 (s, 1H), 7.10 (s, 1H), 7.08 (t, 1H), 7.03 (t, 1H), 6.96 (m, 2H), 4.54 (s, 2H), 4.13 (s, 2H); ^13^C NMR (125 MHz, CD_3_OD): δ 181.46, 171.12, 139.70, 138.08, 123.37, 122.51, 120.86, 119.85, 119.33, 112.24, 111.62, 109.78, 107.89, 25.42, 23.21; HR-ESIMS: [M − H]^−^ calcd. for C_20_H_1__4_ClN_4_O 361.0862, found: 361.0864.

#### 3.1.8. Synthesis of Compound **14l**−**14o**

To a solution of acyl chloride (2.7 mmol, 1.0 equiv) and potassium carbonate (3.2 mmol, 1.2 equiv) in toluene (10 mL), compound **12** (2.7 mmol, 1.0 equiv) was added and under reflux for 4h. The mixture was filtered, and the residue was washed with CH_2_Cl_2_; then, the solution was combined and concentrated. The residue was subjected to silica gel chromatography with petroleum ether/EtOAc (5:1) to give compounds **14l**−**14o**.

**14l:** White solid, Yield 60%; ^1^H NMR (400 MHz, CD_3_OD): δ 7.53 (d, 1H), 7.33 (d, 1H), 7.17 (s, 1H), 7.09 (t, 1H), 6.98 (t, 1H), 4.16 (s, 2H), 3.75 (m, 1H), 2.39 (m, 4H), 2.08 (m, 2H); ^13^C NMR (125 MHz, CD_3_OD): δ 183.61, 171.03, 138.09, 128.33, 124.48, 122.54, 119.86, 119.30, 112.29, 109.77, 32.52, 27.98, 23.22, 19.57; HR-ESIMS: [M + H]^+^ calcd. for C_15_H_16_N_3_O 254.1288, found: 254.1285.

**14m:** White solid, Yield 65%; ^1^H NMR (400 MHz, CD_3_OD): δ 7.53 (d, 1H), 7.33 (d, 1H), 7.17 (s, 1H), 7.09 (t, 1H), 6.98 (t, 1H), 4.16 (s, 2H), 3.75 (m, 1H), 2.39 (m, 4H), 2.08 (m, 2H); ^13^C NMR (125 MHz, CD_3_OD): δ 183.61, 171.03, 138.09, 128.33, 124.48, 122.54, 119.86, 119.30, 112.29, 109.77, 32.52, 27.98, 23.22, 19.57; HR-ESIMS: [M + H]^+^ calcd. for C_16_H_18_N_3_O 268.1382, found: 268.1391.

**14n:** White solid, Yield 60%; ^1^H NMR (400 MHz, CD_3_OD): δ 7.53 (d, 1H), 7.33 (d, 1H), 7.16 (s, 1H), 7.09 (t, 1H), 6.99 (t, 1H), 4.16 (s, 2H), 2.95 (m, 1H), 2.03 (m, 2H), 1.80 (m, 2H); 1.59 (m, 2H); 1.42 (m, 2H); 1.32 (m, 2H); ^13^C NMR (125 MHz, CD_3_OD): δ 184.49, 170.88, 138.12, 128.34, 124.46, 122.54, 119.85, 119.31, 112.29, 109.77, 37.42, 31.25, 30.76, 26.62, 23.34; HR-ESIMS: [M + H]^+^ calcd. for C_17_H_20_N_3_O 282.1601, found: 282.1603.

**14o**: White solid, Yield 63%; ^1^H NMR (400 MHz, CD_3_OD): δ 8.72 (d, 1H), 8.24 (d, *J* = 7.92 Hz, 1H), 8.03 (t, 1H), 7.60 (m, 2H), 7.34 (d, *J* = 8.13 Hz 1H), 7.24 (s, 1H), 7.09 (t, 1H), 7.02 (t, 1H), 4.31 (s, 2H); ^13^C NMR (125 MHz, CD_3_OD): δ 175.36, 172.34, 151.26, 144.35, 139.54, 138.14, 128.45, 125.58, 124.62, 122.59, 119.93, 119.33, 112.33, 109.70, 23.38; HR-ESIMS: [M + H]^+^ calcd. for C_16_H_13_N_4_O 277.1084, found: 277.1089.

### 3.2. Biological Assay

For screening of PTP1B and TCPTP, 2 μL of the stock solution of each compoud (1 mM) in DMSO were transferred into individual wells of 96-well flat bottom plates, to give a final concentration of 20 μM of extract in 2% DMSO. After incubation with the enzymes for 15 min, 10 times-concentrated substrates were added to initiate the enzymatic reaction, and the resultant enzymatic activity normalized against the control (2% DMSO) to obtain the inhibition rate of the compound. When the inhibition rate was more than 50% at 20 μM, the dose–response inhibition assay of the compound was performed to determine the 50% percentage inhibition concentrations (IC_50_).

### 3.3. Molecular Docking

The LigPrep panel was employed to generate stereoisomers and protonation states of our compounds with Epik integrated in Maestro 9.1 (Schrödinger, LLC, New York, NY, USA, 2010) [20,21]. The crystal structure of hPTB1B (PDB access code: 2QBP) was retrieved from the Protein Data Bank (http://www.rcsb.org/pdb/home/home.do) and chosen as the receptor for molecular docking. The Protein Preparation Wizard module integrated in the Maestro program suite was applied to prepare the receptor [22]. Docking simulations were performed using the GLIDE 5.5 (Grid-based Ligand Docking with Energetics) program with the extra precision (XP) mode. Other parameters were set as the default.

## Figures and Tables

**Table 1:** Inhibitory activity of compounds **9a**–**9e** and **10a**–**10e** on PTP1B.

Compd.	R	% Inhibition (20 μM)	Compd.	R	% Inhibition (20 μM)
**9a**	H	7.3 ± 6.8	**10a**	H	2.4 ± 1.2
**9b**	F	14.0 ± 4.3	**10b**	F	19.8 ± 0.1
**9c**	Cl	5.2 ± 1.8	**10c**	Cl	40.0 ± 7.6
**9d**	NO_2_	9.8 ± 7.1	**10d**	NO_2_	44.5 ± 0.3
**9e**	CH_2_CH_3_	23.4 ± 3.7	**10e**	CH_2_CH_3_	50.5 ± 1.1
Phidianidine B (2)		23.5 ± 2.6	Oleanolic acid (IC_50_)		1.2 ± 0.1 μM

**Table 2:** Inhibitory activity of compounds **13a**–**13i** and **14a**–**14u** on PTP1B.

Compd.	X	IC_50_ (μM)	Compd.	X	IC_50_ (μM)
**13a**	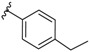	>20	**14a**	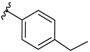	13.5 ± 0.6
**13b**	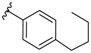	>20	**14b**	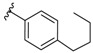	7.1 ± 0.1
**13c**	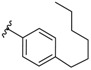	>20	**14c**	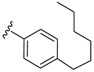	4.9 ± 0.8
**13d**	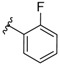	>20	**14d**	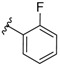	>20
**13e**	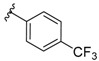	>20	**14e**	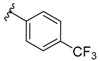	>20
**13f**	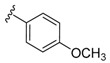	>20	**14f**	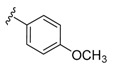	>20
**13g**	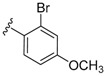	>20	**14g**	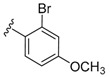	>20
**13h**	(CH_2_)_3_CH_3_	>20	**14h**	(CH_2_)_3_CH_3_	>20
**13i**	(CH_2_)_4_Br	>20	**14i**	(CH_2_)_4_O_2_CCH_3_	>20
**14j**	CH(CH_3_)_2_	>20	**14k**	CH(Cl)_2_	>20
**14l**	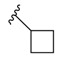	8.6 ± 1.5	**14m**	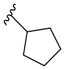	>20
**14n**	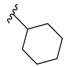	5.3 ± 0.7	**14o**	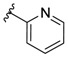	>20
**14p**	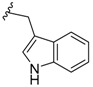	5.8 ± 0.1	**14q**	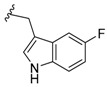	>20
**14r**	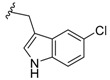	7.9 ± 0.6	**14s**	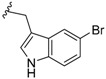	7.4 ± 0.1
**14t**	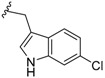	8.7 ± 0.9	**14u**	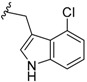	9.7 ± 1.7

**Table 3:** Inhibitory activity of PTP1B inhibitory compounds on T-cell protein tyrosine phosphatase (TCPTP) ^a^.

Compd.	% Inhibition (20 μM)	Compd.	% Inhibition (20 μM)	Compd.	% Inhibition (20 μM)
**10e**	15.84	**14l**	25.52	**14s**	34.51
**14a**	9.22	**14n**	11.68	**14t**	54.35
**14b**	50.31	**14p**	55.17	**14u**	38.74
**14c**	54.18	**14r**	38.29	Oleanolic acid (IC_50_)	3.24 μM

^a^ Compounds possessing IC_50_ values less than 20 μM on PTP1B were evaluated for their TCPTP inhibitory activities.
